# Cell Population Data and Serum Polyclonal Immunoglobulin Free Light Chains in the Assessment of COVID-19 Severity

**DOI:** 10.3390/v13071381

**Published:** 2021-07-15

**Authors:** Milena Małecka-Giełdowska, Maria Fołta, Agnieszka Wiśniewska, Emilia Czyżewska, Olga Ciepiela

**Affiliations:** 1 Department of Laboratory Medicine, Medical University of Warsaw, 02-097 Warsaw, Poland; agnieszka.wisniewska@wum.edu.pl (A.W.); emilia.czyzewska@wum.edu.pl (E.C.); olga.ciepiela@wum.edu.pl (O.C.); 2 Central Laboratory of Central Teaching Hospital, University Clinical Center of Medical University of Warsaw, 02-097 Warsaw, Poland; 3 Students Scientific Group of Laboratory Medicine, Department of Laboratory Medicine, Faculty of Pharmacy, Medical University of Warsaw, 02-097 Warsaw, Poland; mariafolta@wp.pl

**Keywords:** COVID-19, FLC, antibody synthesis lymphocytes

## Abstract

Distinguishing between severe and nonsevere COVID-19 to ensure adequate healthcare quality and efficiency is a challenge for the healthcare system. The aim of this study was to assess the usefulness of CBC parameters together with analysis of FLC serum concentration in risk stratification of COVID-19. Materials and methods: CBC was analyzed in 735 COVID ICU, COVID non-ICU, and non-COVID ICU cases. FLC concentration was analyzed in 133 of them. Results: COVID ICU had neutrophils and lymphocytes with the greatest size, granularity, and nucleic acid content. Significant differences in concentrations of κ and λ FLCs were shown between COVID ICU and COVID non-ICU. However, no difference was found in the κ/λ ratio between these groups, and the ratio stayed within the reference value, which indicates the presence of polyclonal FLCs. FLC κ measurement has significant power to distinguish between severe COVID-19 and nonsevere COVID-19 (AUC = 0.7669), with a sensitivity of 86.67% and specificity of 93.33%. The κ coefficients’ odds ratio of 3.0401 was estimated. Conclusion: It can be concluded that the results obtained from the measure of free light immunoglobulin concentration in serum are useful in distinguishing between severe and nonsevere COVID-19.

## 1. Introduction

Severe acute respiratory syndrome coronavirus (SARS-CoV-2) belongs to the coronavirus family, along with MERS-CoV and SARS-CoV [1]. The virus is transmitted by airborne droplets [2]. It is classified into enveloped viruses, while the genetic material of the virus is single-stranded RNA [3,4]. The first cases of disease caused by SARS-CoV-2 were noted in Wuhan, Hubei, China, in November 2019. Since then, the disease has spread around the world, affecting over 182 million people and causing the death of over 3.9 million of them (worldometer.info/coronavirus, access data 29 June 2021). In February 2020, the disease induced by SARS-CoV-2 was named coronavirus disease 2019 (COVID-19). In March 2020, the World Health Organization announced a global pandemic of COVID-19. Patients infected with SARS-CoV-2 may show the symptoms of fever, dry cough, fatigue, less often muscle pain, sore throat, diarrhea, conjunctivitis, headache, loss of taste or smell, and skin rash. The serious symptoms that manifest the infection are difficulty of breathing or shortness of breath, chest pain or tightness, and loss of speech or motor skills [3]. For SARS-CoV-2 infected patients, the following significant changes in laboratory test results have been demonstrated in previously published manuscripts [5]. During infection, there is an increase in the production and release of inflammatory mediators and proinflammatory cytokines, e.g., interleukin 2 (IL-2), interleukin 6 (IL-6), granulocyte colony-stimulating factor (G-CSF), and tumor necrosis factor α (TNF-α); the concentration of C-reactive protein increases; and the concentration of procalcitonin in infected patients increases 4-fold. The increase in procalcitonin and ferritin in the serum of infected patients is an unfavorable prognostic factor for the patient. In SARS-CoV-2 infection, an increase in the concentration of fibrin decay product (FDP) D-dimer is also observed, which correlates with the advancement of the disease process. Other basic studies monitoring the functioning of the coagulation cascade showed prolongation of both prothrombin (PT) and activated partial thromboplastin (APTT) clotting times [6,7,8]. Furthermore, in infected patients, the levels of FDP and fibrinogen increase as the disease progresses. Patients suffering from COVID-19 have more frequent episodes of disseminated intravascular coagulation (DIC) [9]. Changes were also observed in hematological parameters. Lymphopenia and neutrophilia are frequently observed in patients infected with SARS-CoV-2. The ratio of absolute neutrophils to lymphocytes (NLR) in relation to patients without infection also increases significantly. 

It is essential to broaden the knowledge of the COVID-19 to improve the diagnostic process and enable risk stratification for patients infected by the virus. Free light chains (FLCs) kappa and lambda, like heavy chains, are produced by plasma cells. Light and heavy chains are used by the immune system to create immunoglobulins (Igs) that attack and neutralize the elements that threaten the body, such as bacteria and viruses. Here, we focused particularly on the differentiation of leukocytes and changes in the concentrations of free light chains (FLCs) in serum in patients with COVID-19 who required intensive care or presented less severe symptoms. Our study also included an assessment of inflammatory markers in patients’ sera: C-reactive protein, ferritin, and IL-6.

## 2. Materials and Methods

Our retrospective study was conducted on a group of 735 patients hospitalized at the Central Teaching Hospital of the University Clinical Center of the Medical University of Warsaw. From them, three groups were selected. There were 246 COVID-19 patients hospitalized at the ICU department (COVID ICU, 146 men in age 64 ± 15 and 100 women in age 73 ± 16), 245 COVID-19 patients hospitalized at other hospital units (COVID non-ICU, 130 men in age 62 ± 14 and 115 women in age 57 ± 18), and 244 patients with disorders other than COVID-19 hospitalized at the ICU (non-COVID ICU, 133 men in age 60 ± 19 and 111 women in age 59 ± 17). Hospitalized patients that were suffering from either hematological diseases or renal dysfunction were not qualified to take part in the study. 

The study included the analysis of leukocyte distribution (5-DIFF) based on the results of a complete blood count, for which the Sysmex XN 2000 analyzer was used. Leukocyte differentiation was based both on automatic leukocyte analysis and microscopic assessment of peripheral blood smears from patients infected with the SARS-CoV-2 virus (light microscopy). 

**Figure 1:**
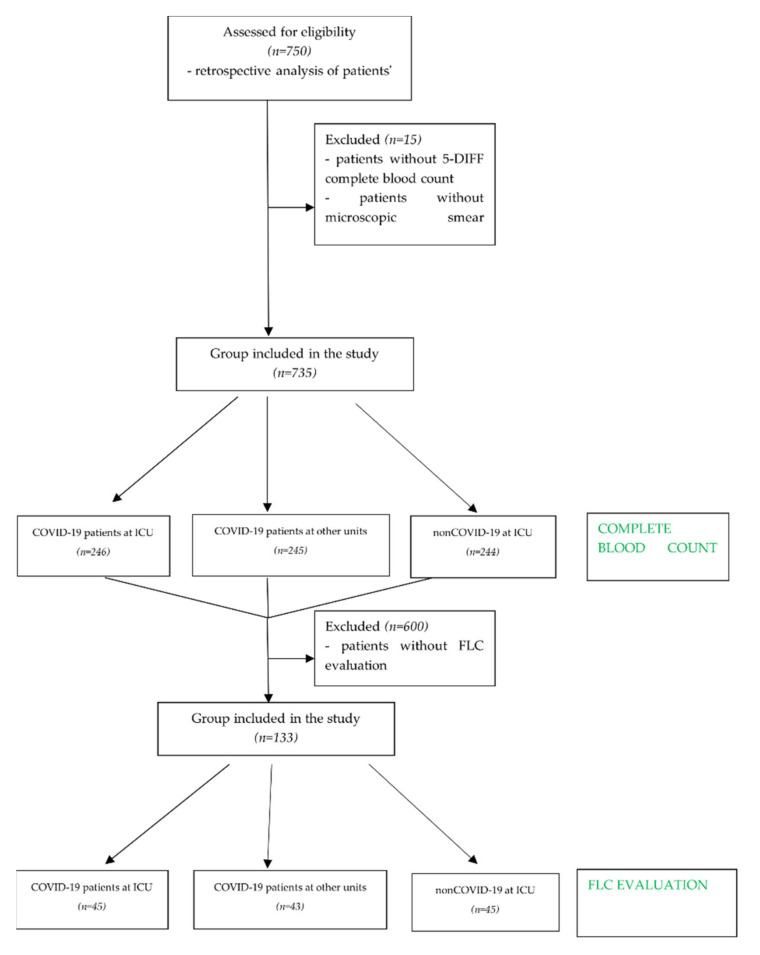
Enrollment scheme—study group selection.

Ninety patients infected with SARS-CoV-2 were tested regarding the presence of free light immunoglobulin chains (FLCs) in serum. To compare FLC serum concentrations between COVID-19 and non-COVID-19 patients who required intensive care, 45 patients were selected from the 245 patients in the non-COVID ICU group, and their serum FLCs were tested. Finally, the FLC study group included 135 subjects. The enrolled study group consisted of 45 patients with COVID-19 hospitalized in the intensive care unit (COVID ICU), 43 patients with COVID-19 hospitalized at other hospital units (COVID non-ICU), and 45 ICU patients in which COVID-19 had not been diagnosed (control group: non-COVID ICU). The COVID-19 ICU patient group was composed of 135 men with a mean age of 63 ± 13.4 and 112 women with a mean age of 81 ± 16.1. The mean age of the COVID-19 non-ICU group was 58 ± 11.5 for men and 62 ± 17.4 for women. The non-COVID ICU group was composed of 135 men, mean age 60 ± 17.39, and 112 women, mean age 61 ± 16.53. From each ICU group (COVID ICU and non-COVID ICU), 45 patients (33 men and 12 women) were selected, and 43 patients (31 men and 12 women) were selected from the COVID non-ICU group; these patients were referred for microscopical smear analysis and assessment of serum FLCs. The inclusion scheme is presented in Figure 1. When selecting a group of patients in order to assess the dependence of the number of lymphocytes synthesizing antibodies and the assessment of light chains in relation to the severity of the disease, the factor selecting the group of patients was the assessment of light chains (FLCs) ordered by a physician in patients hospitalized in our medical unit. 

The Sysmex XN-2000 (Sysmex, Kobe, Japan) analyzer was used to determine complete blood count parameters. As an automatic hematology analyzer, the Sysmex XN-2000 can analyze the complete blood count (CBC) by fluorescence flow cytometry. The WDF measurement channel on the Sysmex XN-2000 analyzer is used to differentiate and count leukocytes based on fluorescence flow cytometry. In the WDF scattergram, cells are classified into individual groups (clusters) according to the size, granularity, and the amount of genetic material (side fluorescence light (SFL), side scatter axes (SSC)). The parameters describing the graphic result of the leukocyte separation are assessed accordingly by WDF-X, leucocyte complexity (leucocyte granularity and amount of genetic material); WDF-Y, leucocyte fluorescence (leucocyte size); WDF-WX, width of dispersion of leucocyte (leucocyte granularity and the amount of genetic material); WDF-WY, width of dispersion of leucocyte fluorescence (leucocyte size); NE-SSC, the lateral scattered light intensity of the NEUT area on the WDF scattergram (neutrophil granularity and the amount of genetic material); NE-SFL, the fluorescent light intensity of the NEUT area on the WDF scattergram (neutrophil size), LY-X, the lateral scattered light intensity of the LYMPH area on the WDF scattergram (lymphocyte granularity and the amount of genetic material); and LY-Y, the fluorescent light intensity of the LYMPH area on the WDF scattergram (lymphocyte size).

The concentration of light chains was determined using the Cobas c501F (Hoffmann-La Roche AG, Basel, Switzerland) analyzer. FLC ĸ and λ concentrations were measured by immunoturbidimetric method on Cobas c501 (Hoffmann-La Roche AG, Basel, Switzerland) using Binding Site Freelite Human Kappa/Lambda. Then, the ratios of ĸ/λ FLCs in serum (reference range 0.26–1.65) were calculated. 

In a retrospective study, the concentrations of IL-6, C-reactive protein, and ferritin were also assessed in the same group of patients infected with SARS-CoV-2 (*n* = 88) in which the concentration of free light chains was determined. IL-6 concentration was measured with the Cobas c801 analyzer (Hoffmann-La Roche AG, Basel, Switzerland) using monoclonal anti-IL-6 antibodies. The tests were performed with the Elecsys IL-6 reagent. Results of C-reactive protein and ferritin concentrations were obtained using the Cobas e702 analyzer (Hoffmann-La Roche AG, Basel, Switzerland). The automatic method for the determination of ferritin is based on the immunological method of agglutination reaction reinforced with latex particles. C-reactive protein was measured with Cobas 702 analyzer (Hoffmann-La Roche AG, Basel, Switzerland) using the latex particle-enhanced turbidimetric method. Human CRP agglutinates with latex particles that are coated with monoclonal antibodies against human C reactive protein. Aggregates are determined using the turbidimetric method.

The statistical analysis of the obtained results was performed in Microsoft Office Excel (Microsoft Corporation, Redmond, WA, USA), MedCalc Software version 19.2.6 (Ostend, Belgium), and GraphPad Prism 9 (GraphPad Software, San Diego, CA, USA). The Mann–Whitney test and Kruskal–Wallis test were employed in the counting and differentiation of leukocytes, determination of the concentration of light chains, and analysis of the graphical results of cell separation. A value of *p* < 0.05 was considered statistically significant for each parameter determined. This retrospective study was accordant to the rules of the Bioethical Committee of the Medical University of Warsaw.

## 3. Results

The results of leukocyte differentiation in non-COVID ICU patients and patients infected with SARS-CoV-2 are presented in Table 1. There were significant differences in almost every study parameter. In general, COVID-19 patients hospitalized in the ICU had the highest neutrophil concentration and the lowest number of lymphocytes, which resulted in the highest NLR ratio. Moreover, the highest number of immature granulocytes was found in the group of COVID-19 subjects hospitalized in the ICU. Interestingly, patients infected with SARS-CoV-2 who were treated in the ICU and those treated in other departments had extremely different numbers of eosinophils: those from ICU had the lowest, and those from non-ICU had the highest eosinophil count from all studied groups (Table 1).

We also evaluated the concentrations of C-reactive protein (CRP), ferritin, and IL-6 in the three groups of patients. It has been shown that the concentration of all inflammatory markers increased significantly in both groups of patients infected with SARS-CoV-2, reaching the highest values in the group of COVID ICU patients (Table 2).

**Figure 2:**
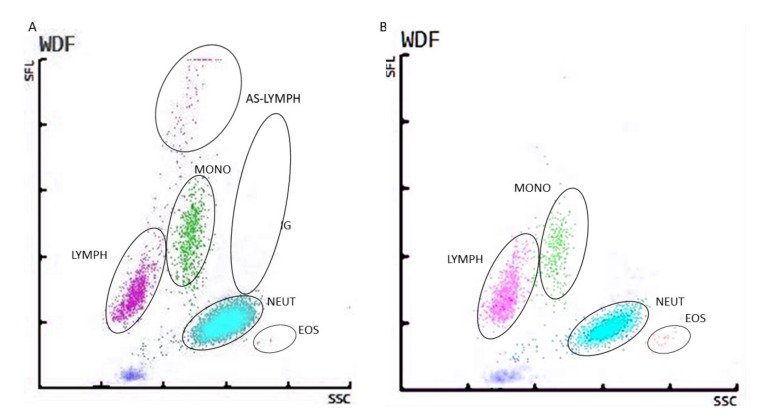
WDF scattergrams of COVID ICU patients (**A**) and non-COVID ICU patients (**B**). Lymph—lymphocytes; Mono—monocytes; Neut—neutrophils; Eos—eosinophils; IG—immature granulocytes; AS-Lymph—antibody-synthesizing lymphocytes.

Automatic analysis of peripheral blood allowed obtaining a WBC differential fluorescence (WDF) scattergram in which the numerical differences in the selected WBC counts were easy to observe (Figure 2).

The scattergram WDF results shown in Figure 2 for COVID ICU (A) and non-COVID ICU (B) include changes in the distribution of leukocyte clusters. For COVID ICU patients, the graph shows a cluster showing the presence of highly fluorescent cells in the blood of infected patients, classified into the group of antibody-synthesizing lymphocytes (AS-Lymph). WDF scattergram of non-COVID ICU patients did not show the presence of AS-Lymph. 

**Figure 3:**
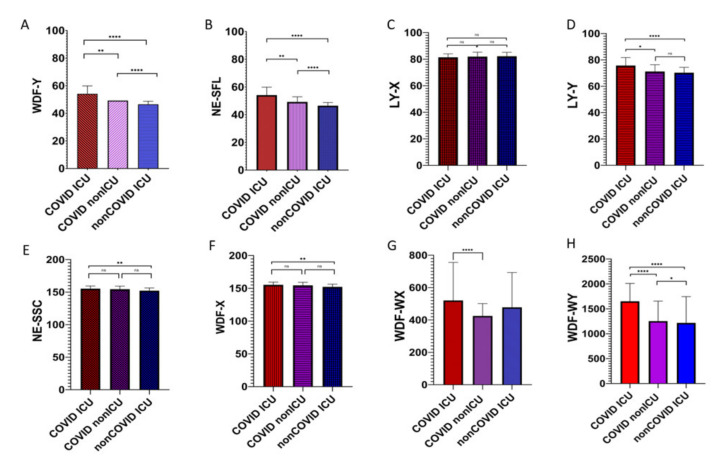
Cell population data (CPD) analysis of graphical results (WDF scattergram) including leukocyte differentiation in three groups: COVID ICU, COVID non-ICU, and non-COVID ICU. A -WDF-Y leucocyte fluorescence (leucocyte size), B - NE-SFL, the fluorescent light intensity of the NEUT area on the WDF scattergram (neutrophils size), C- LY-X, the lateral scattered light intensity of the LYMPH area on the WDF scattergram (lymphocyte granularity and the amount of genetic material), D - LY-Y, the fluorescent light intensity of the LYMPH area on the WDF scattergram (lymphocyte size), E - NE-SSC, the lateral scattered light intensity of the NEUT area on the WDF scattergram (neutrophils granularity and the amount of genetic material), F - WDF-X leucocyte complexity (leucocyte granularity and amount of genetic material), G - WDF-WX width of dispersion of leucocyte (leucocytes granularity and the amount of genetic material), H - WDF-WY width of dispersion of leucocyte fluorescence (leucocytes size); (*n* = 735, *p < 0.05, **p < 0.01, ****p < 0.0001).

The data describing the graphical results obtained with the Sysmex XN-2000 analyzer are based on the following characteristics: the complexity and internal structure of the cell (NE-SSC, LY-X, WDF-WX, WDF-X) and the size and quantity of nucleic acids (NE-SFL, WDF-WY, LY-Y, WDF-Y). We showed statistically significant differences in cell population data (CPD) parameters in our study. The p-values are described in Figure 3. COVID ICU patients had the greatest size, granularity, and nucleic acid content in neutrophils and lymphocytes (WDF-Y, NE-SFL, LY-X, LY-Y, NE-SSC, WDF-X, WDF-WX, and WDF-WY) when compared with other groups. In contrast, patients without SARS-CoV-2 infection hospitalized in the ICU had the lowest values of the mentioned parameters. There was only one parameter (WDF-WX, indicating the granularity of cells analyzed in WDF scattergram) in which there was no difference between COVID ICU and non-COVID ICU, as well as between COVID non-ICU and non-COVID ICU.

In accordance with the results of automatic leukocyte evaluation and change in scattergram, the presence of antibody synthesis lymphocytes in the peripheral blood of COVID-19 patients was indicated. With the increase in leukocytosis, an increasing number of immature granulocytes in blood smears was observed in the group of infected patients (*n* = 45). Pseudo-Pelger–Huët anomaly was observed in a few granulocytes. Toxic granules were observed in the cytoplasm of neutrophils. The presence of erythroblasts was observed in a few smears.

**Figure 4:**
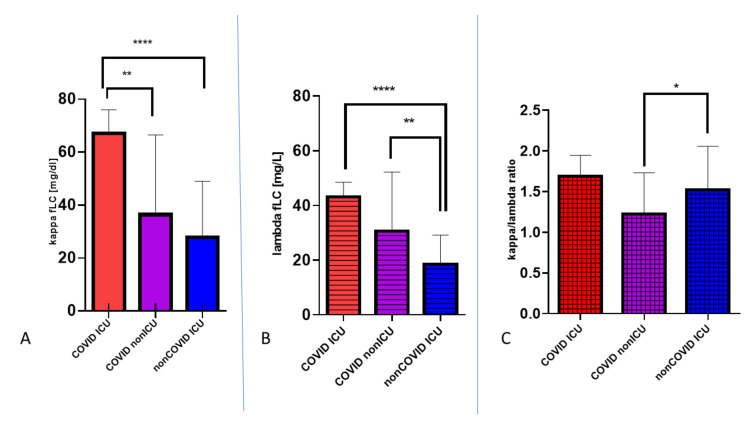
The concentration of FLC chains in three groups of patients (COVID-19 patients hospitalized in the intensive care unit, COVID-19 patients hospitalized in the other units, and non-COVID-19 patients hospitalized in the intensive care unit). A—κ FLCs; B—λ FLCs; C—κ/λ ratio.(* *p* < 0.05, ** *p* < 0.01, **** *p* < 0.0001).

To analyze if the presence of plasmacytoid cells may be associated with increased release of gammaglobulins, the concentrations of the free light chains of immunoglobulins (FLCs) were measured in patients’ sera. Only 45 subjects enrolled in each group were referred for evaluation of the presence of FLCs in serum. There was a significant difference (*p* < 0.0001) in the concentrations of κ and λ FLCs between SARS-CoV-2 infected and noninfected patients hospitalized in the ICU, 67.15 mg/L ± 15.92 mg/L for κ vs. 36.41 mg/L ± 19.74 mg/L for λ, respectively. However, no difference was found in the κ/λ ratio between these two groups, and the ratio stayed within the reference value, which indicates that no production of monoclonal FLCs was found. There was also a significant difference in λ (*p* < 0.01) and κ/λ ratio (*p* < 0.05) between those patients infected with SARS-CoV-2 who did not require intensive care and noninfected patients from the ICU, 43.15 ± 35.23 mg/L vs. 18.44 ± 10.61 mg/L for λ and 1.67 ± 1.65 vs. 1.54 ± 0.58 for κ/λ ratio, respectively. This indicates that, despite severe viral diseases which require hospital admission, the overall response of the immune system is still less intensified in patients not infected with SARS-CoV-2 who require intensive care. (Figure 4).

The results obtained in the study were then analyzed for the correlation of the number of antibody synthesis lymphocytes with the release of free light chains and the FLC ratio (κ FLCs, λ FLCs, κ/λ ratio) in COVID ICU and COVID non-ICU patients. The R^2^ value indicates a very strong correlation between the number of antibody synthesis lymphocytes and the secretion of κ FLCs (R^2^ = 0.995) and λ FLCs (R^2^ = 0.984). No correlation was found between antibody synthesis lymphocyte number and κ/λ ratio in both groups. The results are presented in Table 3. 

Using the logistic regression analysis, we analyzed the odds ratio of patients infected with SARS-CoV-2 in relation to the concentrations of FLCs and the number of antibody synthesis lymphocytes in the peripheral blood (Table 4). The obtained odds ratio for κ light chains took the value of OR = 3.0401, which indicates a 3-fold increased probability of increased synthesis of this type of light chain in patients infected with SARS-CoV-2. 

**Figure 5:**
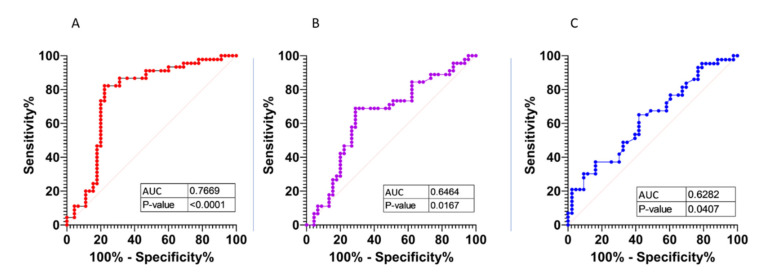
ROC curve analyses of free light chains ((**A**)—ROC curve κ FLCs; (**B**)—ROC curve λ FLCs; (**C**)—ROC curve κ/λ ratio).

We also evaluated if κ, λ, and κ/λ FLC ratio values could distinguish between patients with severe COVID-19 (COVID ICU) and nonsevere COVID-19 (COVID non-ICU) patients. Analysis of ROC curves showed that κ FLCs have the highest power to distinguish between these two groups of patients (AUC = 0.7669), with a sensitivity of 86.67% and specificity of 93.33%. The p-values and AUC are presented in Figure 5. Our study also assessed the correlation of concentration of C-reactive protein with the observed concentrations of κ and λ and the κ/λ FLC ratio. Our observations indicate that there is a correlation between increased CRP, ferritin, and IL-6 concentrations and the increased secretion of FLCs during COVID-19 in the group of patients hospitalized in the ICU (COVID ICU) and in other departments (COVID non-ICU) (Table 5). 

## 4. Discussion

Our study presents an analysis of hematological parameters of leukocyte differentiation as well as the concentration of light immunoglobulin chains and their ratio in patients with severe COVID-19, who required intensive care, those infected with SARS-CoV-2 who did not require intensive care, and the control group of noninfected patients treated in the intensive care unit due to other causes. In the present study, we found that based on cell population data obtained from complete blood analysis and evaluation of FLC concentration, it is possible to distinguish between these three groups of patients. 

Both the changes in the parameters of scattergrams in the control and study group and the changes in the concentration of light immunoglobulin chains are related to the humoral immune response. Here, we show that COVID-19 subjects have lymphopenia and neutrophilia, which is accordant with the observations of other researchers [10,11,12]. 

Tong Mu et al. showed that patients diagnosed with COVID-19 had a reduction in lymphocyte count and percentage and eosinophil count and percentage compared with noninfected patients [13]. Lagunas-Rangel, in his study on the assessment of changes in the neutrophil-to-lymphocyte ratio (NLR) and lymphocyte to C-reactive protein ratio (LCR) in patients with severe coronavirus disease 2019 (COVID-19), showed that NLR is higher in infected patients than in noninfected patients [14]. The results of our study also show that this ratio in the group of patients hospitalized in the intensive care unit suffering from COVID-19 is more than three times higher than that in the group without infection. Interestingly, in our study, we showed that eosinophil count may differ between severe and nonsevere COVID-19. Xie et al., in their manuscript, determined that the number of eosinophils in patients suffering from COVID-19 is reduced. In their analysis, they also linked eosinopenia in patients to the predictive value of this index in conjunction with the parameter of neutrophil-to-lymphocyte ratio (NLR). In the case of a reduced number of eosinophils, their study also showed that this group of patients had a more severe course of the disease than patients with higher numbers of eosinophils in the peripheral blood. They also assessed the utility of this parameter in assessing and monitoring the prognosis of COVID-19 patients. Our analysis confirms the indicated thesis about the possibility of linking the number of eosinophils with a worse course of the disease and the need for hospitalization in the intensive care unit [15]. Progressive eosinopenia in COVID-19 patients was described by Yan et al., who provided information showing that this laboratory parameter correlates with the deteriorating health of infected patients. The mortality risk assessment analyzed by the above-mentioned researchers is also higher in patients with progressive eosinopenia [16]. In the study of Gonzalez et al., the prognostic value of changes in the number of eosinophils and lymphocytes in relation to mortality of patients from the studied cohorts of Spanish patients hospitalized due to SARS-CoV-2 infection was analyzed. A relationship was demonstrated between the number of eosinophils and the mortality rate. Scientists have shown that an increase in the number of peripheral blood eosinophils has a positive effect on prognosis and lowers the mortality rate (OR) [17]. Mao et al., in their study, assessed the effect of the number of eosinophils on the severity of the disease. The researchers reported that the occurrence of eosinopenia negatively affects the prognosis of hospitalized patients [18]. Martens et al. studied the usefulness of hemocytometric parameters for each of the leukocyte groups in patients suffering from COVID-19 who developed or did not develop a cytokine storm. Researchers observed a significant degree of neutrophilia, leukopenia, and thrombocytopenia. The size of the dispersion of lymphocytes (LY-FSC) and reactive lymphocytes (RE-Lymph) reached significantly higher values in COVID-19 subjects with cytokine storm compared to patients without cytokine storm. In our study, there was also a significant difference in hemocytometric parameters between the groups suffering from COVID-19 (COVID ICU and COVID non-ICU) and without infection (non-COVID ICU). Our results also confirm the variable values of neutrophil fluorescence intensity NE-SFL in SARS-CoV-2-infected subjects. Our results seem to confirm that eosinopenia and peripheral blood lymphopenia in patients infected with SARS-CoV-2 may be associated with a cytokine storm resulting from severe viral infection, which requires intensive care [19]. 

Cell population data (CPD), which give information about changes in leukocyte distribution and scattergram parameters in patients hospitalized in the intensive care unit infected and noninfected with SARS-CoV-2, were analyzed in our group of patients. The analysis of changes in cell population distribution parameters during COVID-19 was also performed by Urrechaga et al. Both the above-mentioned study and our results confirm the occurrence of significant changes during COVID-19. However, the utility of CPD parameters is inconsiderable for clinicians because these are only research parameters, available exclusively for laboratory specialists who perform the test. To increase the usefulness of CPD parameters in the diagnosis of SARS-CoV-2 infection, they or their interpretation should be disclosed for the clinicians to suggest an initial diagnosis and then execute the coronavirus infection confirmation test [20]. Changes in CPD have also been studied extensively in patients who developed sepsis and died or not as a result. During systemic infection, an increase in CPD parameters such as NE-SFL and NE-WY appears, which provides us with information about the presence of immature or activated neutrophils, which confirms that these research laboratory parameters may also have clinical applications [21]. We found that in patients with COVID-19, the presence of AS-Lymph clustered with the graphical results. Microscopical examination of peripheral blood smears from COVID-19 patients confirmed that the cluster of AS-Lymph contained antibody synthesis lymphocytes, antibody synthesis lymphocytes, and reactive lymphocytes. This observation could translate into a faster diagnostic course for patients with COVID-19. Medical laboratories that do not have automatic analyzers that can differentiate antibody synthesis lymphocytes from other WBCs could in such cases perform microscopic analysis of the smears or count cells in the smear using automated methods. Leukocyte differentiation in complete blood count and WDF graphic results could contribute to faster preselection of patients with suspected infection. Osman et al., in the analysis of the graphical results, showed a high sensitivity of 82.2% and specificity of 83.5% in relation to the occurrence of a cluster on the WDF scattergram with the presence of antibody synthesis lymphocytes in the complete blood of a patient with COVID-19 [22]. 

The cluster of lymphocytes synthesizing antibodies characteristic for infected patients (AS-Lymph) obtained by an automatic method in the group of our patients and its absence in the control group without infection hospitalized in the same department was verified by the automatic leukocyte count and the reference method—microscopic smear evaluation. As in the studies carried out so far, the presence of antibody synthesis lymphocytes [23] and the presence of toxic granules in neutrophils were observed, and in a few neutrophils, the pseudo-Pelger–Huët anomaly was observed [5]. Unlike other researchers, we did not observed blue-green cytoplasmic inclusions in neutrophils and monocytes [24]. In the study of Mira et al., a left shift was observed in the smear. Band neutrophils and sparse immature granulocytes (metamyelocytes, myelocytes, promyelocytes) have been observed in patients suffering from COVID-19 [25]. Many studies have confirmed the observation of an increase in inflammatory markers in the course of SARS-CoV-2 infection [26].

To search for further parameters useful for risk stratification in COVID-19, our team performed determinations of the concentrations of κ and λ light chains and the κ/λ ratio. Therefore far, an analysis of the concentration of free light chains in patients with COVID-19 has not been performed. Our study provides such results in regard to the correlation of their concentration with survival and odds ratio (OR) of SARS-CoV-2 infected patients hospitalized in intensive care units. 

The differences between the concentrations of kappa and lambda chains in patients from the COVID ICU, COVID non-ICU, and non-COVID ICU groups prove that infection with SARS-CoV-2 stimulates FLC synthesis in the human body, most importantly in the group of COVID-19 patients hospitalized in the intensive care unit. Here, we report that there were also significant differences in the rate of light immunoglobulin chain synthesis during infection, with a significant predominance of κ chains. This is probably related to the rate of gene activation for the kappa light chain reported in the literature. The activation of the gene on chromosome 2 responsible for the synthesis of the kappa chain is slightly faster than the activation of the gene on chromosome 22 responsible for the synthesis of the lambda chain [27].

Excessive production of light chains is observed during hematological disorders with the production of clonal antibody synthesis lymphocytes and after stimulation of the immune system. 

Gudowska-Sawczuk et al. found that in the course of multiple sclerosis and HIV infection there was an increased concentration of light chains in serum and cerebrospinal fluid. Similarly, Presslauer et al. showed that during multiple sclerosis, the level of kappa FLCs significantly increases compared to their level in other diseases of the central nervous system [28]. 

Polyclonal free light chains were also proposed as biomarkers of inflammatory diseases and useful in an evaluation as a treatment target [29]. Many studies also indicate an increase in the concentration of free light chains during autoimmune diseases such as systemic lupus erythematosus [30], rheumatoid arthritis, Sjögren’s syndrome [31], chronic obstructive pulmonary disease, multiple sclerosis, asthma, and food allergies [30,32]. 

During inflammation accompanying many diseases, excessive FLC synthesis occurs in the patient’s body. Here, we report that the synthesis of kappa chains significantly increases as the infection with SARS-CoV-2 progresses. Our results are in line with the results of other studies regarding FLC synthesis in viral diseases. In the course of chronic hepatitis C, the serum levels of FLCs were also increased, with an accompanying increase in the κ/λ ratio. Moreover, high FLC concentration was associated with mixed cryoglobulinemia, B-cell non-Hodgkin lymphoma, and amyloidosis [33,34]. Likewise, in HIV infection, increased concentrations of κ FLCs predict diagnosis of B-cell lymphoma [35].

Kumar et al. assessed the usefulness of the determination of the level of light chains in patients with a median age of 18.1 years. They showed that an increase in FLC levels is correlated with the underlying inflammation, which predisposes patients to develop monoclonal gammopathy of undetermined significance (MGUS) [36]. 

In the course of a cytokine storm during SARS-CoV-2 infection, the current reports indicate an increase in the concentration of IL-6 and ferritin in a patient’s serum. The increase in the secretion of IL-6 is observed in the course of reactions related to the acute phase of inflammation occurring in the course of trauma, stress, infections, cerebral death, and neoplastic processes. Chen et al. investigated changes in laboratory parameters in the course of a COVID-19-induced cytokine storm. In their study, they included information on a significant increase in the concentration of IL-6 and ferritin compared to the reference ranges for these parameters [37]. Sabaka et al. evaluated the predictive value of IL-6 determination in the course of COVID-19 in the assessment of the severity of the disease. The researchers’ analysis of the ROC curve showed that IL-6 is the most powerful predictor of hypoxemia. On the other hand, the concentration of IL-6 above 24 pg/mL indicated the development of hypoxemia with a sensitivity of 100% and a specificity of 88.9% [38]. Parviz et al. found that the level of IL-6, the level of ferritin, and hematological parameters including WBC and the number of lymphocytes and neutrophils had a direct positive correlation with the concentration of hemoglobin [39]. Our observations are in line with their results. However, the study performed on our patients indicates significantly higher concentrations of ferritin and IL-6 in patients with COVID-19 requiring intensive care comparing with those hospitalized in non-ICU departments. With our results, we confirm observations made by other groups showing that IL-6 allows distinguishing between severe and nonsevere COVID-19 [38,39,40,41,42].

Thus, monitoring of FLC concentration in COVID-19 convalescents might be essential for early detection of possible development of lymphoproliferative disorders.

## 5. Conclusions

In conclusion, our research may contribute to broadening the knowledge about the influence of SARS-CoV-2 infection on the parameters of leukocyte differentiation. The comparison of leukocyte counting methods, which have not been developed so far in the group of patients hospitalized in intensive care units, will contribute to an increase in the number of verified automatic complete blood count results in the case of patients with COVID-19. The assessment of the production of an increased amount of light chains in the patient’s body, especially kappa-type chains, may be a predictive factor in assessing the severity of COVID-19 and future outcome.

## Figures and Tables

**Table 1:** Comparison of the results of leukocyte differentiation in patients infected with SARS-CoV-2 and noninfected (*n* = 735).

		COVID-19 Patients Hospitalized in the Intensive Care Unit	COVID-19 Patients Hospitalized in the other Units	Non-COVID-19 Patients Hospitalized in the Intensive Care Unit	
Parameter	SI Units	Mean	Mean	Mean	*p*-Value
WBC *(n = 735)*	(10^9^/L)	11.42 (10.42–12.41)	9.18 (8.23–10.13)	7.64 (7.18–8.15)	*p^1^ < 0.0001* *p^2^ < 0.0001* *p^3^ < 0.0001*
NEUT *(n = 735)*	(10^9^/L)	8.97 (7.96–9.94)	6.31 (5.55–7.02)	4.68 (4.24–5.16)	*p^1^ <0.0001* *p^2^ < 0.0001* *p^3^ < 0.0001*
NEUT *(n = 735)*	(%)	73.0 (71.0–75.0)	66.96 (64.6–69.4)	57.3 (55.3–59.1)	*p^1^ < 0.0001* *p^2^ < 0.0001* *p^3^ < 0.0001*
LYMPH *(n = 735)*	(10^9^/L)	1.33 (1.21–1.45)	1.60 (1.43–1.86)	1.95 (1.79–2.11)	*p^1^ <0.0001* *p^2^ < 0.0001* *p^3^ < 0.0001*
LYMPH *(n = 735)*	(%)	15.40 (13.80–16.90)	19.61 (17.78–21.43)	28.5 (26.83–30.12)	*p^1^ < 0.0001* *p^2^ < 0.0001* *p^3^ < 0.0217*
MONO *(n = 735)*	(10^9^/L)	0.85 (0.71–0.99)	0.91 (0.69–1.13)	0.71 (0.66–0.76)	*p^1^ < 0.0001* *p^2^ < 0.0001* *p^3^ < 0.0001*
MONO *(n = 735)*	(%)	7.9 (7.3–8.6)	9.4 (8.5–10.3)	9.9 (8.5–10.3)	*p^1^ = 0.0001* *p^2^ = 0.0001* *p^3^ = 0.0001*
EOS *(n = 735)*	(10^9^/L)	0.10 (0.07–0.12)	0.12 (0.09–0.15)	0.20 (0.16–0.23)	*p^1^ < 0.0001* *p^2^ < 0.0001* *p^3^ < 0.0001*
EOS *(n = 735)*	(%)	1.20 (0.88–1.46)	1.49 (1.12–1.87)	2.8 (2.35–3.17)	*p^1^ < 0.0001* *p^2^ < 0.0001* *p^3^ < 0.0001*
BASO *(n = 735)*	(10^9^/L)	0.04 (0.03–0.05)	0.07 (0.06–0.22)	0.04 (0.02–0.04)	*p^1^ < 0.0001* *p^2^ < 0.0001* *p^3^ < * *0.0001*
BASO *(n = 735)*	(%)	0.3 (0.3–0.4)	0.9 (0.9–2.5)	0.6 (0.6–0.7)	*p^1^ < 0.0001* *p^2^ < 0.0001* *p^3^ < 0.0001*
IG *(n = 735)*	(10^9^/L)	0.3 (0.2–0.4)	0.2 (0.1–0.4)	0.1 (0.05–0.1)	*p^1^ < 0.0001* *p^2^ < 0.0001* *p^3^ < 0.0001*
IG *(n = 735)*	(%)	2.0 (1.2–2.8)	1.70 (1.4–2.0)	0.9 (0.5–1.3)	*p^1^ < 0.0001* *p^2^ < 0.0001* *p^3^ < 0.0001*
NLR *(n = 735)*		10.25 (8.85–11.63)	8.28 (7.13–9.43)	3.97 (2.78–5.21)	*p^1^ < 0.0001* *p^2^ < 0.0001* *p^3^ = 0.0079*

WBC—leucocyte count; NEUT—neutrophils; LYMPH—lymphocytes; MONO—monocytes; EO—eosinophils; BASO—basophils; IG—immature granulocytes; NLR—neutrophil/lymphocyte ratio; *p*^1^—*p*-values COVID ICU vs. COVID non-ICU; *p*^2^—*p*-values COVID ICU vs. non-COVID ICU; *p*^3^—*p*-values COVID non-ICU vs. non-COVID ICU.

**Table 2:** Comparison of the results of C-reactive protein (CRP), ferritin, and IL-6 concentrations in patients infected with SARS-CoV-2 and noninfected.

		COVID-19 Patients Hospitalized in the Intensive Care Unit	COVID-19 Patients Hospitalized in the other Units	Non-COVID-19 Patients Hospitalized in the Intensive Care Unit
CRP *(n = 735)*	Mean (mg/L)	145.7	85.2	34.9
	95% CI	115.9–175.4	57.9–112.5	24.4–45.5
	*p*-value	*p* < 0.001	*p* < 0.001	*p* < 0.001
Ferritin *(n = 88)*	Mean (μg/L)	2178	518.0	112.6
	95% CI	1765 to 2591	280.5 to 755.4	76.29 to 148.9
	*p*-value	*p* < 0.0001	*p* < 0.0001	*p* < 0.0001
IL-6 *(n = 88)*	Mean (pg/mL)	2203	85.15	33.52
	95% CI	1323 to 3083	50.72 119.6	10.41 to 77.44
	*p*-value	*p* < 0.0001	*p* < 0.0001	*p* < 0.0001

**Table 3:** Correlation of the light chains with the presence of antibody synthesis lymphocytes in a blood smear (*n* = 88).

Parameter	COVID-19 Patients Hospitalized in Intensive care Units		COVID-19 Patients Hospitalized in Other Units		*p*-Value	R^2^
	Mean	95% CI for the Mean	Mean	95% CI for the Mean		
κ (mg/L)	47.03	43.52 to 64.76	24.62	21.22 to 36.45	*p*^1^ = 0.0020 *p*^2^ < 0.0001	0.995
λ (mg/L)	34.71	30.66 to 47.23	25.83	19.26 to 28.38	*p*^1^ = 0.0167 *p*^2^ < 0.001	0.984
κ/λ	1.34	1.1990 to 1.5150	1.27	1.06 to 1.35	*p*^1^ = 0.1108 *p*^2^ = 0.4017	
mean of antibody synthesis lymphocytes in manual smear	6		2			

*p*^1^—*p*-values COVID ICU vs. COVID non-ICU; *p*^2^—*p*-values COVID ICU vs. non-COVID ICU.

**Table 4:** Odds ratio of the severity of the disease related to hospitalization of patients in the intensive care unit (COVID ICU) (*n* = 88).

Parameter	OR	95% CI
κ (mg/L)	3.0401	0.1592 to 58.0000
λ (mg/L)	0.9956	0.9075 to 1.0956
κ/λ	0.9879	0.931 to 1.0482
antibody synthesis lymphocytes	0.0930	0.0091 to 0.9332

**Table 5:** Correlation of the increased C-reactive protein, ferritin, and IL-6 concentrations with the increased secretion of free light chains during COVID-19 (*n* = 88).

		κ (mg/L)	λ (mg/L)	κ/λ	CRP (mg/L)	Ferritin (μg/L)	IL-6 (pg/mL)	*p*-value	R^2^
COVID ICU	Mean	47.03	34.71	1.34	147.5	2178	2203	*p*^1^ < 0.0001 *p*^2^ < 0.0001 *p*^3^ < 0.0001	0.986 0.845 0.978
	95% CI for the mean	43.52 to 64.76	30.66 to 47.23	1.20 to 1.52	116.0 to 179.0	1765 to 2591	1323 to 3083		
COVID non-ICU	Mean	24.62	25.83	1.27	67.15	518.0	85.15	*p*^1^ < 0.0005 *p*^2^ < 0.0001 *p*^3^ < 0.0001	0.876 0.990 0.889
	95% CI for the mean	21.22 to 36.45	19.26 to 28.38	1.06 to 1.35	50.32 to 98.07	280.5 to 755.4	50.72 119.6		

*p*^1^—*p*-value of C-reactive protein concentration in COVID ICU patients in conjunction with secretion of free light chains; *p*^2^—*p*-value of ferritin concentration in COVID ICU patients in conjunction with secretion of free light chains; *p*^3^—*p*-value of IL-6 concentration in COVID ICU patients in conjunction with secretion of free light chains; *p*^1^′—*p*-value of C-reactive protein concentration in COVID ICU patients in conjunction with secretion of free light chains; *p*^2^′—*p*-value of ferritin concentration in COVID ICU patients in conjunction with secretion of free light chains; *p*^3^′—*p*-value of IL-6 concentration in COVID ICU patients in conjunction with secretion of free light chains.

## Data Availability

The data presented in this study are available on request from the corresponding author. The data are not publicly available due to ethical reasons.
